# Will the climate of plant origins influence the chemical profiles of cuticular waxes on leaves of *Leymus chinensis* in a common garden experiment?

**DOI:** 10.1002/ece3.5930

**Published:** 2019-12-17

**Authors:** Yang Li, Xiangyang Hou, Xiaoting Li, Xiao Zhao, Zinian Wu, Yu Xiao, Yanjun Guo

**Affiliations:** ^1^ College of Agronomy and Biotechnology Southwest University Chongqing China; ^2^ Chinese Academy of Agricultural Science Institute of Grassland Research Hohhot China

**Keywords:** adaptation, common garden experiment, intraspecific trait variation, leaf wax, *Leymus chinensis*

## Abstract

Cuticular wax covering the leaf surface plays important roles in protecting plants from biotic and abiotic stresses. Understanding the way in which plant leaf cuticles reflect their growing environment could give an insight into plant resilience to future climate change. Here, we analyzed the variations of cuticular waxes among 59 populations of *Leymus chinensis* in a common garden experiment, aiming to verify how environmental conditions influence the chemical profiles of cuticular waxes. In total, eight cuticular wax classes were identified, including fatty acids, aldehydes, primary alcohols, alkanes, secondary alcohols, ketones, β‐diketones, and alkylresorcinols, with β‐diketones the predominant compounds in all populations (averaged 67.36% across all populations). Great intraspecific trait variations (ITV) were observed for total wax coverage, wax compositions, and the relative abundance of homologues within each wax class. Cluster analysis based on wax characteristics could separate 59 populations into different clades. However, the populations could not be separated according to their original longitudes, latitudes, annual temperature, or annual precipitation. Redundancy analysis showed that latitude, arid index, and the precipitation from June to August were the most important parameters contributing to the variations of the amount of total wax coverage and wax composition and the relative abundance of wax classes. Pearson's correlation analysis further indicated that the relative abundance of wax classes, homologues in each wax class, and even isomers of certain compound differed in their responses to environmental factors. These results suggested that wax deposition patterns of *L. chinensis* populations formed during adaptations to their long‐term growing environments could inherit in their progenies and exhibit such inheritance even these progenies were exported to new environments.

## Graphical abstract

We analyzed the variations of cuticular waxes among 59 populations of *Leymus chinensis* in a common garden experiment. Great intraspecific trait variations (ITV) were observed for total wax coverage, wax compositions and the relative abundance of homologues within each wax class. Our results suggested that wax deposition patterns of *L. chinensis* populations could inherit in their progenies.

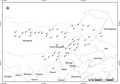

## 1 INTRODUCTION

Plants are sessile organisms, which have evolved the abilities to capture and utilize resources under changing environments. During this evolution process, plastic variations of functional traits enable the plants to survive through different growth conditions and to exhibit adaptive differentiation of plant populations in response to differing climates (van Kleunen & Fischer, 2005). The cuticle, covering leaf surface, plays important roles in protecting plants from biotic and abiotic stresses (Yeats & Rose, 2013). Understanding the way in which plant leaf cuticles reflect their environment could give an insight into plant resilience to future climate change.

Cuticular waxes are mixtures of hydrophobic compounds, which include the epicuticular wax covering on the cutin surface and the intracuticular wax embedded within the cutin matrix (Jeffree, 2006). Though the wax amount and composition vary between plant species, different organs, growing stages, and even growing conditions, most plants share similar wax biosynthesis pathways (Samuels, Kunst, & Jetter, 2008). The main wax chemicals identified in plants are long‐chain fatty acids and their derivatives such as aldehydes, primary alcohols, alkanes, alkyl esters, ketones, secondary alcohols, and triterpenoids (Jetter, Kunst, & Samuels, 2006). Studies have shown that plants will alter their wax deposition and compositions under changing environments to improve their adaptations (Hatterman‐Valenti, Pitty, & Owen, 2011; Mackova et al., 2013; Shepherd & Griffiths, 2006). For example, greater weighed mean alkane chain length at low and at high elevations was possibly a result of adaptation to minimize cuticular permeability due to high summer temperature at low elevation and freezing causing physiological drought at high elevations (Dodd & Poveda, 2003). An increase of alkane deposition has been observed in many drought stressed plants (Gefen, Talal, Brendzel, Dror, & Fishman, 2015). Dodd, Rafii, and Power (1998) also reported that annual rainfall was the most significant factor in regressions with the shorter‐chain hydrocarbons whereas annual mean temperature was most significant for the longer‐chain hydrocarbons. These reports implied that plant populations growing under different environments might exhibit intraspecific variations of wax profiles.

Plant species can display high intraspecific trait variation (ITV) under different growing conditions, reflecting heritable genetic variation and phenotypic plasticity (Henn et al., 2018). The variations between populations may be attributed to the differences in the selective pressures imposed by different ecological environments (Still, Kim, & Aoyama, 2005). Understanding the patterns of functional trait variation across environmental gradients offers an opportunity to increase inference in the mechanistic causes of plant community assembly (Fajardo & Siefert, 2018). Based on an analysis from more than 2,500 vascular plant species, Wright et al. (2005) found that 18% of variation in their study of leaf traits was explained by climate. Climate influences trait variation in part by selection for different life forms and families, and the trait values derived from climate data via redundancy analysis (RDA) showed substantial predictive power for trait values in the available global datasets (Yang et al., 2019). In a climate gradient in the east side of Qinghai‐Tibetan Plateau, Guo, Guo, He, and Gao (2015) found that the mean annual temperature and aridity index were significantly correlated with the averaged amounts of wax compositions and total leaf cuticular wax. Many studies have also validated that the intraspecific variation of leaf cuticular wax could be applied in distinguishing plant populations growing under different environments (Bojovic et al., 2012; Halinski, Szafranek, & Stepnowski, 2011). Therefore, understanding ITV of leaf cuticular wax across regional scales can illustrate the potential adaptive capacity of plant populations to local conditions.

In addition to the large amount of information on the wax compound identifications, the relationship between wax and abiotic stresses, and gene cloning involved in wax biosynthesis, little is known in revealing genetic differentiation of plant leaf cuticular waxes from different regions and populations. In order to seek evidence for an adaptive response in wax composition and quantity across environmental and geographic gradients, Ramirez‐Herrera, Percy, Loo, Yeates, and Vargas‐Hernandez (2011) found that strong differentiation among regions and populations within regions was observed for wax quantity from 12 *Pinus pinceana* populations in a common garden test. Based on a study of *Melaleuca quinquenervia* across eastern Australia, Andrae et al. (2019) found that *n*‐alkane characteristics were not a plastic response to climate variability and instead were likely fixed and could be driven by genetic differences between populations. Using quantitative genetic and quantitative trait loci analyses, Gosney et al. (2016) also reported that the variation and differentiation in cuticular wax compounds within *Eucalyptus globulus* had a complex genetic origin. Common garden experiments are a powerful tool to test genetic variations among populations. Selection of plant species that are widely distributed across various environments and exhibit obvious ITV provides us the possibility to reveal the genetic differentiation of plant leaf cuticular waxes.

*Leymus chinensis* (Trin.) Tzvel, a perennial forage and ecological grass, is widely distributed throughout the eastern end of the Eurasian steppe, including western North Korea, Mongolia, and the north western part of Siberia and is centered in north eastern China (Yuan, Ma, Guo, & Wang, 2017). In China, it grows across diverse soil and climate conditions such as the Songliao Plain, the Inner Mongolia Plateau, and the Loess Plateau, contributing to its wide genetic diversity (Liu, Li, Li, Yang, & Liu, 2007). In order to determine whether cuticular wax in *L. chinensis* exhibits similar or different patterns of local adaptation across their distributions, in this study, we selected 59 populations distributed in different environments, aiming to verify how environmental conditions could influence the chemical profiles of cuticular waxes in *L. chinensis* in a common garden experiment. In addition, the relationship between meteorological variables and the qualitative and quantitative wax compositions was explored in order to detect meteorological factors affecting wax profiles. We hypothesized that intraspecific variability in leaf wax production and chain length distributions of *L. chinensis* might be genetic differentiation of plant leaf cuticular waxes.

## 2 METHODS

### 2.1 Site description

**Figure 1:**
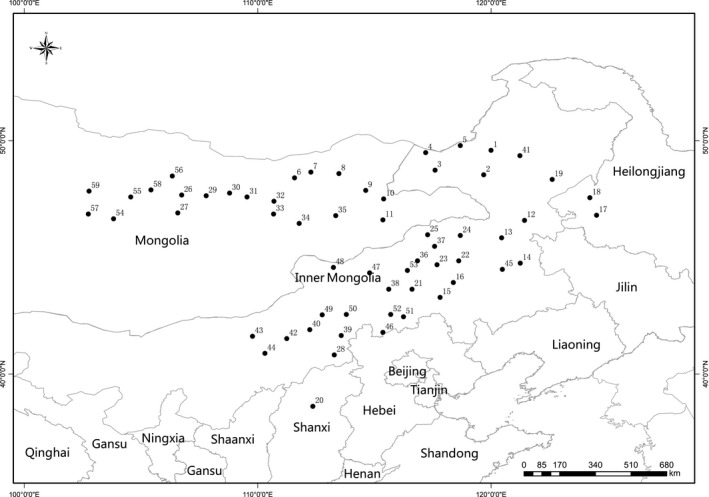
Geographic distribution of collection sites for the *Leymus chinensis* populations used in this study. Latitude and longitude data are from https://www.latlong.net/. Each number represented one sampling site

We established a common garden experiment design, located in Shaerqin research station (N40°34′, E111°56′) of the Institute of Grassland Research, Chinese Academy of Agricultural Science, Hohhot, Inner Mongolia, China. The altitude of this station is 1,065 m. The annual temperature and annual rainfall were 6.3°C and 440 mm across last 30 years. The soil type was calcic Kastanozem (FAO), with pH of 8.3. The contents of soil total organic carbon, total nitrogen, available nitrogen, available phosphorus, and potassium were 16.7 g/kg, 1.09 g/kg, 69.45 mg/kg, 20.5 mg/kg, and 124 mg/kg. In total, 59 *L. chinensis* populations, rhizomes of which were collected from different environmental conditions (Figure 1, Table 1), were grown in a common garden environment, minimizing plastic responses to the environments. Each population was planted in a 4 m × 4 m plot using rhizomes collected in each environmental condition in 2013, and the aboveground parts were harvested two times each year from 2014, one in early July and one in September when the plants were in their maturities. There were about 200 plants in each plot. The plots were weeded whenever other plant species appeared and fertilized in May each year with 20 kg N, 15 kg P_2_O_5_, and 15 kg K when plants started to regrow.

**Table 1:** Climate factors of the sites where the *Leymus chinensis* populations were collected. The climate data were the average across 30 years from 1996 to 2016

Site	Longitude	Latitude	*T*_a_	*T*_6_	*T*_7_	*T*_8_	*P*_a_	*P*_678_	Arid index
1	120.0	49.6	−0.8	18.1	20.4	18.2	330.1	230.1	36.0
2	119.7	48.5	−0.9	18.0	20.3	18.1	327.9	226.8	36.0
3	117.6	48.7	1.6	19.6	22.0	19.8	244.6	177.2	21.1
4	117.2	49.5	1.6	19.6	22.0	19.8	244.6	177.2	21.1
5	118.7	49.8	−0.8	18.1	20.4	18.2	330.1	230.1	36.0
6	111.6	47.4	−0.1	16.7	18.8	16.7	199.4	137.7	20.1
7	112.3	48.7	−0.1	16.7	18.8	16.7	282.8	200.4	28.4
8	113.5	48.6	−0.5	15.0	16.2	14.5	282.8	200.4	29.8
9	114.6	47.9	0.7	17.6	19.8	17.9	282.8	200.4	26.5
10	115.4	47.5	0.7	17.9	20.5	18.3	190.5	123.9	17.8
11	115.4	46.6	0.7	17.9	20.5	18.3	190.5	123.9	17.8
12	121.4	46.6	2.9	18.2	20.5	18.6	449.1	330.3	34.8
13	120.5	45.8	6.7	21.7	23.8	22.4	361.9	265.9	21.6
14	121.3	44.8	7.1	21.9	24.1	22.6	361.9	270.9	21.2
15	117.8	43.3	3.2	17.9	20.5	18.6	391.7	258.9	29.6
16	118.4	43.9	5.8	20.3	22.6	20.9	361.8	265.0	23.0
17	124.5	46.8	3.5	20.3	22.8	20.9	419.9	295.7	31.2
18	124.2	47.6	3.5	20.3	22.8	20.9	442.9	308.9	32.9
19	122.6	48.3	3.7	19.6	21.7	19.8	501.2	369.2	36.7
20	112.4	38.6	9.6	21.6	23.3	21.8	427.4	269.9	21.8
21	116.6	43.6	3.0	19.2	21.6	19.8	263.5	179.8	20.2
22	118.6	44.9	2.0	17.6	20.2	18.5	324.9	217.4	27.0
23	117.7	44.7	2.0	17.6	20.2	18.5	324.9	217.4	27.0
24	118.7	45.9	0.3	17.2	19.9	18.2	320.9	222.7	31.2
25	117.3	46.0	1.9	18.8	21.4	19.7	251.0	182.7	21.0
26	106.8	47.7	0.7	17.6	19.8	17.9	207.6	139.7	19.5
27	106.6	46.9	4.3	20.3	22.0	20.3	110.0	74.8	7.7
28	113.3	40.8	5.6	19.0	21.0	19.0	363.5	235.0	23.4
29	107.8	47.6	−2.2	14.4	16.2	14.3	216.4	150.9	27.7
30	108.8	47.8	2.1	19.4	20.9	18.5	223.5	157.0	18.4
31	109.6	47.6	2.1	19.4	20.9	18.5	223.5	157.0	18.4
32	110.7	47.4	−0.6	16.4	18.5	16.5	245.0	212.0	26.2
33	110.7	46.9	−0.6	16.4	18.5	16.5	223.5	157.0	23.9
34	111.8	46.5	1.4	17.6	20.5	18.4	111.3	78.0	9.7
35	113.4	46.8	−1.0	16.4	18.2	16.3	204.4	147.1	22.7
36	116.9	44.9	2.0	17.6	20.2	18.5	324.9	217.4	27.0
37	117.6	45.5	1.9	18.8	21.4	19.7	251.0	182.7	21.0
38	115.6	43.6	1.9	18.7	21.3	19.3	238.1	166.4	20.0
39	113.6	41.7	4.2	18.0	20.1	18.2	337.1	217.9	23.8
40	112.2	41.9	4.0	18.0	20.3	18.1	315.2	191.6	22.5
41	121.2	49.4	−1.8	16.6	19.1	17.0	388.5	254.9	47.3
42	111.3	41.5	2.9	17.2	19.4	17.1	280.2	180.4	21.7
43	109.8	41.6	4.7	19.4	21.7	19.3	251.8	164.0	17.1
44	110.3	40.9	5.3	19.8	21.9	19.4	291.1	184.9	19.1
45	120.5	44.5	7.1	21.9	24.1	22.6	361.9	270.9	21.2
46	115.4	41.8	2.4	16.4	18.7	16.9	383.4	236.0	30.9
47	114.8	44.3	1.2	18.0	20.6	18.6	224.4	159.1	20.0
48	113.3	44.6	3.6	20.1	22.8	20.7	180.4	117.6	13.3
49	112.8	42.5	5.5	20.4	23.0	20.8	201.6	128.9	13.0
50	113.8	42.6	3.9	18.6	21.1	19.0	270.6	166.8	19.5
51	116.3	42.5	2.5	17.0	19.4	17.5	359.6	230.9	28.7
52	115.7	42.6	2.8	17.5	19.9	17.9	351.2	217.4	27.5
53	116.4	44.4	2.0	17.6	20.2	18.5	324.9	217.4	27.0
54	103.8	46.7	0.8	14.0	15.3	13.9	199.4	137.7	18.5
55	104.6	47.6	−1.8	13.0	14.5	12.8	369.7	262.9	45.0
56	106.4	48.5	−1.0	16.4	18.2	16.3	282.8	200.4	31.5
57	102.8	46.9	−1.1	13.4	14.6	12.8	216.4	150.9	24.2
58	105.4	47.9	−2.4	14.6	16.6	14.7	216.4	150.9	28.5
59	102.8	47.8	0.2	13.0	14.3	12.8	258.1	178.9	25.4

Note

*T*_a_, annual average temperature; *T*_6_, *T*_7_, and *T*_8_ represented average temperature in June, July and August; *P*_a_, annual precipitation; *P*_678_, rainfall from June to August.

### 2.2 Sampling

In July 2016, when plants were in their heading stages, leaves (the third leaf from the top) were sampled from each plant population separately. There were no strict biological replicates for each population, thus leaves from 10 plants were mixed and regarded as one replicate, in total three replicates for each population. To avoid the difference of wax deposition in different plant development stages, we sampled plants only in heading stages. The leaves were washed gently in water to exclude dusts on leaf surface and stored in absorbent papers. The absorbent papers were changed every other day until the leaves were fully dried without going moldy.

### 2.3 Cuticular wax extraction

Due to the difficulties in extracting the wax from fresh leaves in fields, dried leaves were used to extract cuticular waxes. In a pre‐experiment study, the dried leaves were extracted in chloroform for 30s, 50s, 60s, and 80s. The results showed that 60s was sufficient to extract most of the cuticular waxes on plant leaves. Therefore, in this study, leaves were extracted in 50 ml chloroform containing 25 µg tetracosane as internal standard at room temperature for 1 min. The extracts were dried in a nitrogen stream at 40°C, and derivated using 50 µl of BSTFA (*N*,*O*‐Bis(trimethylsilyl) Trifluoro Acetamide) and 50 µl pyridine (Aldrich) for 45 min at 70°C. The surplus solutions were evaporated under nitrogen, and the sample was redissolved in 200 µl chloroform for GC and GC/MS analysis.

For compound identification, the GC analysis was carried out with 9790Ⅱ gas chromatograph (Fu‐Li, China). The GC column was DM‐5 capillary column (30 m × 0.32 mm × 0.25 µm). Nitrogen was served as the carrier gas. The GC oven was held at 80°C for 10 min and heated at 5°C/min to 260°C, where the temperature remained 10 min. The temperature was then heated at 2°C/min to 290°C and further heated at 5°C/min to 320°C, where the temperature was held for 10 min. Compounds were detected with a GCMS‐QP2010 Ultra Mass Spectrometric Detector (Shimadzu Corp.) using HP‐5 MS capillary column (30 m × 0.32 mm × 0.25 µm) and He as the carrier gas. Compounds were identified by comparing their mass spectra with published data and authentic standards.

Quantification was based on FID peak areas. After wax extraction, the surface areas of leaves were measured with a WinFOLIA professional leaf image analysis system (Regent Instrument, Inc.) and digitizing scanner (EPSON V750, Japan). The amount of wax was expressed in µg/cm^2^.

### 2.4 Data analysis

In order to evaluate whether properties of the site of origin of an accession affected its fitness in the novel environment of Shaerqin, historical climate data for the collection sites of the accessions were obtained from National Meteorological Information Center of China (http://data.cma.cn/). Latitude and longitude for all of the accessions were obtained from https://www.latlong.net/. For each site, the weather information from the grid point closet to the historical site of origin of the accession was used. An aridity index was calculated as *I* = *P*/(*T* + 10), where P is the annual precipitation in mm and T is the mean annual temperature in degrees centigrade (Dodd & Poveda, 2003). In order to evaluate whether chemical compositions varied between populations, one‐way ANOVA was applied on total wax coverage (SPSS 18.0). Hierarchical cluster analysis was applied based on amounts of total wax coverage and wax compositions, the relative abundance of wax compositions, and the relative abundance of compound homologues within each wax class, according to furthest neighbor (SPSS 18.0). Redundancy analysis was further undertaken to visualize the relationship between climate factors and the amounts of total wax coverage and wax compositions, the relative abundance of wax compositions, and the relative abundance of compound homologues within each wax class, using R software (3.6.1) and the vegan and ggplot2 package.

## 3 RESULTS

### 3.1 Variations of cuticular wax among ecotypes

**Figure 2:**
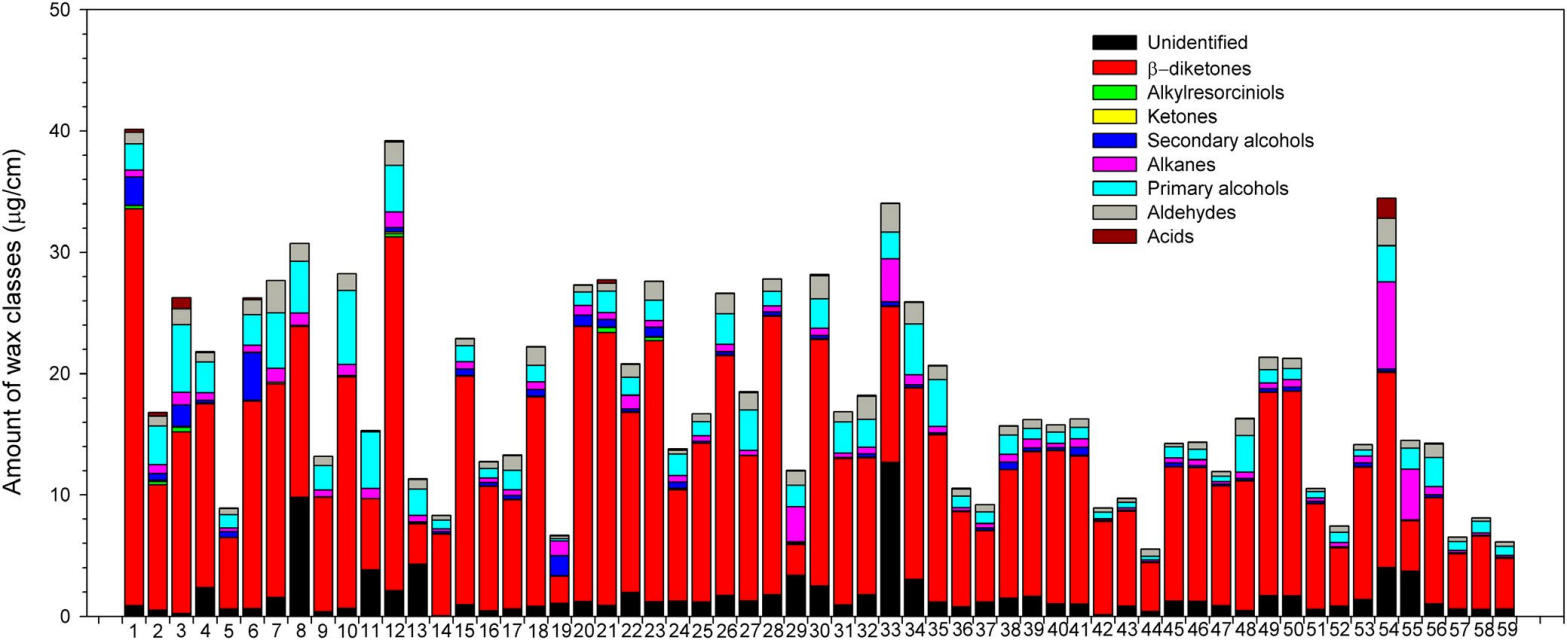
Variations of leaf cuticular wax classes among 59 populations of *Leymus Chinensis* in a common garden experiment

In total, eight cuticular wax classes were identified from 59 populations, including fatty acids, aldehydes, primary alcohols, alkanes, secondary alcohols, ketones, β‐diketones, and alkylresorcinols (Table 2). The number of populations that acids had not been detected was 10, secondary alcohol was 7, ketone was 6, and alkylresorcinol was 50 (Figure 2). Diketone was the dominant compound class in *L. chinensis*, accounting for 21.36% to 84.64% in total wax, followed by primary alcohol (averaged 10.63% across all population), aldehydes (5.22%), and alkanes (4.53%). The total wax ranged from 5.55 µg/cm^2^ to 40.14 µg/cm^2^, with coefficient of variance reaching 46.82%. ANOVA analysis further indicated that the amount and the relative abundance of wax compositions varied greatly between populations in a common garden experiment, showing high ITV (Table 3).

**Table 2:** Variations of the amounts and relative abundance of wax compositions across 59 *Leymus chinensis* populations

Composition	Amount (µg/cm^2^)				Relative abundance (%)			
	Min.	Max.	Ave.	CV	Min.	Max.	Ave.	CV
Acids	0.00	1.67	0.09	268.19	0.00	4.03	0.41	171.65
Aldehydes	0.12	2.64	0.93	63.03	0.76	12.71	5.22	44.79
Primary alcohols	0.18	6.13	1.90	71.60	2.74	30.25	10.63	54.80
Alkanes	0.13	7.20	0.82	135.80	1.43	28.04	4.53	110.40
Secondary alcohols	0.00	3.93	0.40	160.28	0.00	26.73	2.32	170.55
Ketones	0.00	0.15	0.04	85.88	0.00	0.78	0.21	79.82
Diketones	2.23	32.74	12.39	52.15	21.36	84.64	67.36	22.16
Alkylresorcerols	0.00	0.39	0.03	273.65	0.00	1.55	0.15	263.48
Unidentified	0.05	12.71	1.68	123.43	0.69	34.72	9.18	84.85
Total wax	5.55	40.14	17.90	49.59				

Abbreviations: Ave., average; CV, coefficient of variance; Min., minimum; Max., maximum.

**Table 3:** ANOVA analysis of the amounts and the relative abundance of wax compositions across 59 *Leymus chinensis* populations

Composition	Amounts			Relative abundance		
	*df*	*F*‐value	*p*	*df*	*F*‐value	*p*
Acids	58	2.262	<.0001	58	4.056	<.0001
Aldehydes	58	6.011	<.0001	58	4.075	<.0001
Primary alcohols	58	11.712	<.0001	58	7.271	<.0001
Alkanes	58	1.490	.035	58	2.813	<.0001
Secondary alcohols	58	10.064	<.0001	58	6.943	<.0001
Ketones	58	4.058	<.0001	58	6.02	<.0001
Diketones	58	9.503	<.0001	58	12.517	<.0001
Alkylresorcerols	58	19.409	<.0001	58	36.163	<.0001
Unidentified	58	5.727	<.0001	58	16.992	<.0001

Each compound class was consisted of series of compounds with varying chain lengths (Table 4). Acids, primary alcohols, and aldehydes contained even carbons, whereas alkanes included both even and odd carbons. The amount and the relative abundance of these homologues also varied greatly between populations. The chain length of acids ranged from 24 (trace) to 30, with the predominant compound varied among populations. The chain length of aldehydes ranged from 26 to 32, with C28 the predominant homologue in most populations. The chain length of alkanes ranged from 25 to 33, with C31 the predominant homologue in most populations. The chain length of primary alcohols ranged from 26 to 34, with C_28_ the predominant homologue in most populations. The secondary alcohol included C_29_ and C_31_ homologues, showing great variations among populations (CV of C_29_ reached 527.53%). Two homologues of alkylresorcinols were observed, with alkane chain length ranging between 21 and 23. The diketones observed in *L. chinensis* were consisted of C_29_ and C_31_ homologues, with the functional groups on carbon 14 and 16 and C_31_ hydroxylated on carbon 6. The predominant homologue of diketone varied among populations, with the CV ranged from 17.96% to 90.74%.

**Table 4:** Variations of the relative abundance of homologues within each wax class across 59 *Leymus chinensis* populations

Compositions	Min.	Max.	Ave.	CV (%)	*df*	*F*‐value	*p*
Hexacosanoic acid	0.00	100	60.24	82.22	58	145.87	<.0001
Octacosanoic acid	0.00	61.30	6.01	279.07	58	233.66	<.0001
Triacontanoic acid	0.00	37.85	2.61	315.16	58	52.76	<.0001
Hexadecanal	0.00	4.21	0.26	327.41	58	28.52	<.0001
Octacosal	17.08	100	61.95	26.42	58	29.16	<.0001
Triacontanal	0.00	73.36	15.41	60.46	58	25.29	<.0001
Dotriacontanal	0.00	57.52	21.18	60.23	58	22.38	<.0001
Hexacosanol	0.00	25.88	2.93	123.21	58	206.08	<.0001
Octacosanol	54.57	100	91.48	21.06	58	30.57	<.0001
Triacontanol	0.00	43.83	4.87	204.68	58	2.08	.0004
Dotriacontanol	0.00	11.48	0.88	207.76	58	119.92	<.0001
Tetratriacontanol	0.00	1.42	0.03	531.66	58	63.52	<.0001
Pentacosane	0.00	29.22	4.11	101.71	58	4.01	<.0001
Hexacosane	0.00	51.35	4.34	162.02	58	3.62	<.0001
Heptacosane	0.00	25.71	7.65	66.00	58	17.30	<.0001
Octacosane	0.00	85.93	5.15	231.24	58	12.11	<.0001
Nonacosane	0.00	50.65	20.92	48.65	58	4.32	<.0001
Triacontane	0.00	63.98	7.01	174.03	58	39.13	<.0001
Hentriacontane	0.00	88.41	37.00	41.80	58	22.48	<.0001
Tritriacontane	0.00	28.58	13.99	46.79	58	36.78	<.0001
14,Hydroxylated‐ nonacosanol	0.00	100	3.37	527.53	58	15,105.07	<.0001
6,Hydroxylated‐ Tritriacontanol	0.00	78.80	4.06	343.32	58	118.29	<.0001
7,Hydroxylated‐ Tritriacontanol	0.00	100	81.32	48.64	58	55.35	<.0001
Heneicosylresorcinol	0.00	100	6.16	310.79	58	257.98	<.0001
Tricosylresorcinol	0.00	100	8.59	278.67	58	421.32	<.0001
Nonacosane(C_29_)−14,16‐dione	0.00	10.90	1.83	90.74	58	165.69	<.0001
Hentriacontane(C_31_)−14,16‐dione	0.00	70.01	34.24	32.59	58	9.72	<.0001
OH‐Hentriacontane(C_31_)−14,16‐dione	19.08	97.76	64.04	17.96	58	8.09	<.0001

### 3.2 Relationship among populations

**Figure 3:**
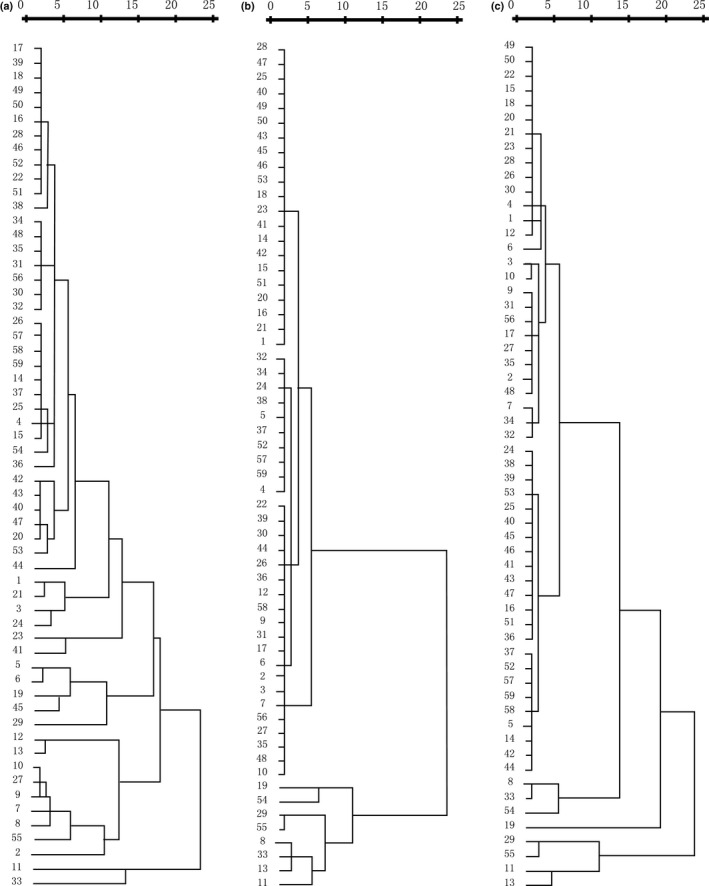
Hierarchical cluster analysis (Furthest neighbor) of 59 *Leymus chinensis* populations based on amounts of total wax coverage and wax compositions (a), the relative abundance of wax compositions (b), and the relative abundance of compound homologues within each wax class (c)

UPGMA cluster analysis, based on wax amounts, relative abundance of wax compositions and the relative abundance of homologues in each wax class, separated 59 populations into two clades (Figure 3). Population 11 and 13 could be separated from most populations using wax amount and relative abundance, whereas population 11, 13, 29, and 55 could be separated from most populations using the relative abundance of wax class and the relative abundance of wax homologues. Most of the populations grouped together and could not separate them according to their geographical distributions.

**Figure 4:**
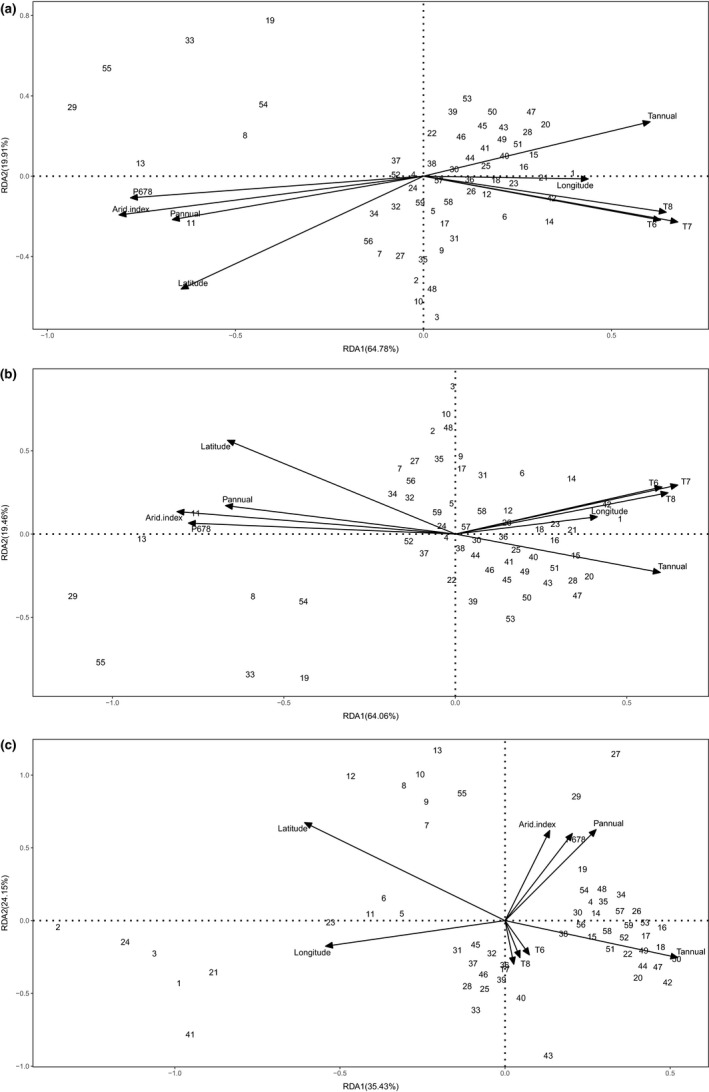
Redundancy analysis (RDA) on data of wax and environmental factors based on amounts of total wax coverage and wax compositions (a), the relative abundance of wax compositions (b), and the relative abundance of compound homologues within each wax class (c). Tannual, annual average temperature; *T*_6_, *T*_7_, and *T*_8_ represented average temperature in June, July and August; Pannual, annual precipitation; *P*_678_, rainfall from June to August

Redundancy analysis revealed the effects of climate factors on the wax characteristics. RDA1 and RDA2 explained 64.78% and 19.91%, respectively, of the changes of the amount of total wax coverage and wax composition (Figure 4a), explained 64.06% and 19.46% of the changes of the relative abundance of wax class (Figure 4b), and 35.43% and 24.15% of the relative abundance of wax homologues (Figure 4c). RAD1 was positively correlated with longitude and annual average temperature, and negatively correlated with latitude, annual precipitation, and arid index, for RDA based on the amount of total wax coverage and wax composition and the relative abundance of wax class. RDA2 was positively correlated with annual average temperature and negatively correlated with all other factors, for RDA with the amount of total wax coverage and wax composition, and negatively correlated with annual average and positively correlated with all other factors, for RDA with the relative abundance of wax class. RDA also showed that latitude, arid index, and the precipitation from June to August, which explained 8.05%–12.18% of variance (*p* = .005), were the most important parameters contributing to the amount of total wax coverage and wax composition and the relative abundance of wax class (Table 5).

**Table 5:** The rates of total variance of wax characteristics explained by the environmental factors from redundancy analysis in Figure 4

	Longitude	Latitude	*T*_a_	*T*_6_	*T*_7_	*T*_8_	*P*_a_	*P*_678_	Arid index
A	3.78	8.05*	5.32	5.94	7.07	6.23	6.96	9.53*	10.64*
B	3.58	8.10*	4.89	5.31	6.38	5.62	6.74	9.16*	10.29*
C	1.34	3.22*	1.49	0.53	0.40	0.36	1.73*	1.49	1.41

Note

A, based on amounts of total wax coverage and wax compositions; B, the relative abundance of wax compositions; C, the relative abundance of compound homologues within each wax class. *T*_a_, annual average temperature; *T*_6_, *T*_7_ and *T*_8_ represented average temperature in June, July and August; *P*_a_, annual precipitation; *P*_678_, rainfall from June to August.

* Significance at *p* = .005.

### 3.3 Relationship between wax amount and the climate factors

Pearson's correlation analysis was applied to further test the influence of climate factors on wax properties (Table 6). There was no significant relationship between total wax coverage and the environmental factors. However, the relative abundance of wax compositions in total wax and the relative abundance of wax homologues in each wax class showed positive or negative correlation with environmental factors. For example, the longitude was positively correlated with the relative abundance of secondary alcohols and alkylresorcinols, whereas the latitude was positively correlated with the relative abundance of ketones and primary alcohols and negatively correlated with the relative abundance of β‐diketones. Annual average temperature was negatively correlated with the relative abundance of alkane and primary alcohols and positively correlated with β‐diketone, whereas the annual precipitation and the rainfall during June to August were positively correlated with the relative abundance of secondary alcohols and β‐diketones and negatively correlated with primary alcohols. Positive correlation was observed between the arid index and the relative abundance of alkanes, secondary alcohols, and alkylresorcinols. The relative abundance of wax homologues differed in their relationship with environmental factors. For example, the longitude was positively correlated with C_26_ and C_32_ primary alcohols and negatively correlated with C_28_ primary alcohol, annual precipitation, the rainfall during June to August, and the arid index were positively correlated with C_26_ and C_30_ primary alcohol and negatively correlated with C_28_ primary alcohol. For β‐diketone, the longitude and annual precipitation were negatively correlated with C_31_ diketone and positively correlated with C_31_ hydroxylated diketone.

**Table 6:** Spearman's correlation coefficient between climate factors and the relative abundance of wax compositions and the relative abundance of homologue in each wax class

	Longitude	Latitude	*T*_a_	*T*_6_	*T*_7_	*T*_8_	*P*_a_	*P*_678_	Arid index
Total wax	−0.013	0.171	−0.069	0.023	0.015	0.007	−0.166	−0.167	−0.144
Acids	0.137	0.137	−0.053	0.074	0.075	0.086	−0.042	0.005	0.071
Aldehydes	−0.245	0.290*	−0.171	−0.116	−0.145	−0.161	−0.231	−0.197	−0.044
Primary alcohols	−0.152	0.543**	−0.465**	−0.207	−0.196	−0.251	−0.433**	−0.350**	−0.067
Alkanes	0.141	0.251	−0.258*	−0.224	−0.200	−0.189	0.145	0.173	0.315*
Secondary alcohols	0.496**	−0.058	0.110	0.225	0.223	0.255	0.419**	0.437**	0.330*
Ketones	0.106	0.333*	−0.078	−0.019	−0.055	−0.044	0.190	0.242	0.234
Alkylresorcinols	0.451**	0.184	−0.098	0.099	0.095	0.114	0.227	0.252	0.264*
Diketones	0.178	−0.532**	0.471**	0.302*	0.292*	0.329*	0.307*	0.244	−0.069
Hexacosanoic acid	−0.030	−0.153	0.215	0.127	0.154	0.202	−0.048	−0.070	−0.098
Octacosanoic acid	0.237	0.166	−0.181	0.007	0.005	0.015	−0.007	0.023	0.127
Triacontanoic acid	0.224	0.226	−0.220	0.026	0.014	−0.009	−0.035	0.002	0.120
Hexadecanal	−0.091	0.306*	−0.247	−0.228	−0.239	−0.262*	−0.161	−0.146	0.006
Octacosal	−0.003	0.247	−0.195	−0.086	−0.077	−0.082	−0.089	−0.029	0.015
Triacontanal	−0.061	−0.112	0.141	0.080	0.098	0.088	−0.240	−0.258*	−0.228
Dotriacontanal	−0.001	−0.307*	0.296*	0.163	0.119	0.145	0.240	0.198	0.053
Hexacosanol	0.326*	−0.233	0.152	0.061	0.048	0.087	0.414**	0.391**	0.343**
Octacosanol	−0.406**	−0.049	−0.025	−0.065	−0.037	−0.059	−0.505**	−0.491**	−0.468**
Triacontanol	0.236	−0.077	0.159	0.186	0.191	0.198	0.385**	0.362**	0.229
Dotriacontanol	0.325*	0.275*	−0.160	0.063	0.036	0.007	0.062	0.118	0.151
Tetratriacontanol	0.181	0.176	−0.181	0.026	−0.009	−0.062	0.101	0.119	0.225
Pentacosane	0.102	0.094	−0.095	0.097	0.081	0.051	−0.097	−0.089	−0.116
Hexacosane	−0.070	−0.069	−0.125	−0.167	−0.161	−0.175	−0.145	−0.181	−0.036
Heptacosane	0.170	0.095	−0.077	0.054	0.048	0.043	−0.054	−0.049	−0.027
Octacosane	0.011	−0.223	0.173	0.017	0.008	0.045	0.268*	0.209	0.118
Nonacosane	0.015	0.248	−0.141	−0.143	−0.165	−0.133	0.072	0.115	0.139
Triacontane	0.130	−0.474**	0.429**	0.212	0.230	0.264	0.279*	0.212	−0.030
Hentriacontane	−0.060	−0.010	0.123	0.073	0.079	0.084	−0.074	−0.077	−0.051
Tritriacontane	0.096	0.027	0.066	0.085	0.063	0.071	0.118	0.147	0.078
14,Hydroxylated‐ nonacosanol	0.323*	0.161	0.043	0.205	0.184	0.172	0.188	0.219	0.135
6,Hydroxylated‐ Tritriacontanol	0.262*	0.234	−0.331*	−0.094	−0.086	−0.085	0.043	0.066	0.274**
7,Hydroxylated‐ Tritriacontanol	−0.138	−0.372**	0.298*	0.119	0.121	0.136	−0.003	−0.031	−0.191
Heneicosylresorcinol	0.401**	0.155	−0.031	0.173	0.157	0.155	0.190	0.235	0.205
Tricosylresorcinol	0.414**	0.212	−0.190	0.008	0.005	0.027	0.202	0.211	0.309*
Nonacosane(C_29_)‐14,16‐dione	−0.007	−0.102	0.122	−0.029	−0.039	−0.003	0.042	0.089	0.031
Hentriacontane(C_31_)‐14,16‐dione	−0.317*	0.205	−0.257*	−0.125	−0.124	−0.148	−0.401**	−0.353**	−0.251
OH‐Hentriacontane(C_31_)‐14,16‐dione	0.305*	−0.176	0.241	0.127	0.123	0.143	0.387**	0.336**	0.247

Note

*T*_a_, annual average temperature; *T*_6_, *T*_7_ and *T*_8_ represented average temperature in June, July and August; *P*_a_, annual precipitation; *P*_678_, rainfall from June to August.

* indicates *p* < .05.

** indicates *p* < .01.

## 4 DISCUSSION

A number of studies have demonstrated the responses of cuticular waxes to changing environments on many plant species. For example, drought stress would increase wax deposition (particularly alkanes) on leaf and thus reduce water loss through leaf cuticle (Gonzalez & Ayerbe, 2010), and enhanced UV‐B irradiation would alter the crystal structures of epicuticular wax and thus increase the reflection of irradiation from leaf (Gordon, Percy, & Riding, 1998). Shepherd, Robertson, Griffiths, Birch, and Duncan (1995) also reported that epicuticular waxes from outdoor‐grown plants were found to have higher proportions of *n*‐alkanes, octacosanoic acid, primary alcohols, and long‐chain esters but lower proportions of aldehydes, ketones, ketols, and secondary alcohols than waxes from indoor‐grown plants. Such responses of plant cuticular waxes have been shown to be related to plant adaptations to changing environments (Yeats & Rose, 2013).

In this study, high ITV was observed among populations of *L. chinensis* for total wax coverage, wax compositions, and the relative abundance of wax homologues within each wax class when tested in a common garden experiment. This suggested that plant populations growing under certain environment might alter leaf wax deposition to adapt to their growing environment, and such adaptation might be inherited and continues to be present when the plants are exported to new environments. A study on *Arabidopsis thaliana* has also shown that the climate histories of the accessions were better predictors of performance than many of life history and growth measures taken during the experiment in a common garden experiment (Rutter & Fenster, 2007). As the outmost of plant surface, cuticular waxes are directly contacting with the atmosphere. A study on *M. quinquenervia* has also shown that plants have evolved their specific wax characteristics to adapt to their surroundings (Andrae et al., 2019). Based on UPGMA cluster analysis, the plant populations could be separated into different clades using wax characteristics. This suggested that cuticular wax, as chemotaxonomy indicator, could be used to analyze the phylogenetic diversity of *L. chinensis*. For example, cuticular hydrocarbons were proposed as potential chemotaxonomic markers in the classification of tomato and related species (Haliński et al., 2015), and *n*‐alkane distribution was useful for species characterization and establishment of links among Malpighiaceae species (Motta, Salatino, & Salatino, 2009), and cluster analysis based on the pattern of the *n*‐alkane distribution allowed to clearly separate the populations of *Plantago major* according to the average annual temperature of their habitats (Guo, He, Guo, Gao, & Ni, 2015). However, the cluster based on wax characteristics could not distinguish the *L. chinensis* populations according to their longitudes, latitudes, annual temperatures, or the sampling transects (Figure 1). For example, cluster analysis based on the relative abundance of wax compositions in total wax coverage and the relative abundance of homologues within each wax class separated population 11, 13, 29, and 55 from most of the other populations, whereas the annual average temperature of the places where these population were originated was 0.7, 6.7, −2.2, and −1.8°C. On one hand, the localization of the imported plant population in common garden experiment might have already changed the wax deposition pattern of *L. chinensis*, differing from their original wax characteristics. This is reasonable because plants have to adjust their wax deposition to adapt to the new environments. In a reciprocal transplant experiment, Knight and Miller (2004) reported that small‐scale local adaptation might be more likely in clonal *Hydrocotyle bonariensis* plants undergoing little gene flow in spatially heterogeneous environments. In a study with *Claytonia perfoliata*, McIntyre and Strauss (2014) concluded that fixed differences in trait values corresponding to selection across habitats contribute to local adaptation, but that plasticity and maternal environmental effects may be favored through promotion of survival across heterogeneous environments. On the other hand, environmental factors influencing wax deposition are complicated. Besides the climate factors as we obtained, soil water conditions as well as UV‐B irradiation in their original places might also influence the wax deposition pattern (Gordon et al., 1998; Schwab et al., 2015), and thus the cluster results.

To further analyze how the climate factors influenced the wax characteristics, RDA analysis and Pearson's correlation analysis were applied. RDA showed that latitude, arid index, and the precipitation from June to August, which explained 8.05% to 12.18% of variance, were the most important parameters contributing to the amount of total wax coverage and wax composition and the relative abundance of wax class. Positive or negative correlation between wax characteristics and longitudes and latitudes was also observed in Pearson's correlation analysis. These results suggested that the current characteristics of the cuticular wax were the responses to comprehensive environmental factors (Jeffree, 2006), which might partly could explain the comprehensive cluster results based on cuticular wax among different populations. This further implied that current leaf wax characteristics partly could reflect paleo‐environmental conditions during which period the plant population formed and grew. For example, alkane C‐23 as a robust proxy for *Sphagnum mosses* was used in paleoecological studies (Bush & McInerney, 2013), and aeolian‐derived higher‐plant lipids in the marine sedimentary record were linked with paleoclimate (Poynter, Farnimond, Robinson, & Eglinton, 1989).

In this study, the relative abundance of wax classes differed in their responses to climate factors. For example, annual temperature was negatively correlated with the relative abundance of primary alcohols and alkanes but positively correlated with β‐diketone, whereas annual precipitation was positively correlated with the relatively abundance of secondary alcohol but negatively correlated with primary alcohols. This implied that historical growing environments influenced the wax biosynthesis pathways in plant populations, resulting in the changes of wax proportions, which might further contribute to plant adaptations (Shepherd, Robertson, Griffiths, & Birch, 1997; Yeats & Rose, 2013). Meanwhile, the relative abundance of wax homologues in each wax class and even the isomers of wax compound also differed in their responses to climate factors. For example, the annual precipitation was positively correlated with the relative abundance of C_26_ and C_30_ primary alcohol but negatively correlated with C_28_ primary alcohol, and negatively correlated with C_31_ β‐diketone but positively correlated with hydroxylated C_31_ β‐diketone. This further implied that the homologue genes involved in wax biosynthesis might also differ in their sensitivity to climate factors, contributing to increased adaptability to diverse environments during plant evolution and domestication process (Zou et al., 2012).

In conclusion, the great ITV of wax characteristics among 59 populations of *L. chinensis* in a common garden experiment indicated that plant populations growing under certain environment might inherit their specific leaf wax deposition patterns to progenies. Such trait inheritance includes total wax coverage, wax compositions, and the chain length distribution patterns. RDA analysis showed that latitude, arid index, and the precipitation from June to August were the most important parameters contributing to the variations of the amount of total wax coverage and wax composition and the relative abundance of wax class. Pearson's correlation analysis further indicated that the relative abundance of wax class, homologues in each wax class, and even isomers of certain compound differed in their responses to environmental factors, suggesting that genes involved in wax biosynthesis showed heterogeneous evolution process in different environments, which contributes to the plant adaptations to growing environments.

## CONFLICT OF INTEREST

The authors declare that they have no conflict of interest.

## AUTHOR CONTRIBUTIONS

LY, LX, ZX, and XY collected the samples and analyzed the GC data and climate data; WZ managed the common garden experiment; HX and GY designed the experiment; GY identified the wax compounds. All authors contributed to the drafts of the manuscript and its final approval.

## Data Availability

We agree to make our data publicly available in a relevant repository (DRYAD, https://doi.org/10.5061/dryad.v15dv41s5) when the manuscript was accepted, including climate factors, wax amount, and wax class abundance.
